# Photosynthetic characteristics and chloroplast ultrastructure of welsh onion (*Allium fistulosum* L.) grown under different LED wavelengths

**DOI:** 10.1186/s12870-020-2282-0

**Published:** 2020-02-17

**Authors:** Song Gao, Xuena Liu, Ying Liu, Bili Cao, Zijing Chen, Kun Xu

**Affiliations:** 1 College of Horticulture Science and Engineering, Shandong Agricultural University, Tai’an, China; 2 Collaborative Innovation Center of Fruit & Vegetable Quality and Efficient Production in Shandong, Tai’an, China; 3 Key Laboratory of Biology and Genetic Improvement of Horticultural Crops in Huanghuai Region, Ministry of Agriculture and Rural Affairs, Tai’an, People’s Republic of China; 4 State Key Laboratory of Crop Biology, Tai’an, 271018 China

**Keywords:** Light, Photosynthetic characteristics, Chloroplast ultrastructure, Welsh onion (*Allium fistulosum* L.)

## Abstract

**Background:**

The optimized illumination of plants using light-emitting diodes (LEDs) is beneficial to their photosynthetic performance, and in recent years, LEDs have been widely used in horticultural facilities. However, there are significant differences in the responses of different crops to different wavelengths of light. Thus, the influence of artificial light on photosynthesis requires further investigation to provide theoretical guidelines for the light environments used in industrial crop production. In this study, we tested the effects of different LEDs (white, W; blue, B; green, G; yellow, Y; and red, R) with the same photon flux density (300 μmol/m^2^·s) on the growth, development, photosynthesis, chlorophyll fluorescence characteristics, leaf structure, and chloroplast ultrastructure of Welsh onion (*Allium fistulosum* L.) plants.

**Results:**

Plants in the W and B treatments had significantly higher height, leaf area, and fresh weight than those in the other treatments. The photosynthetic pigment content and net photosynthetic rate (*P*_n_) in the W treatment were significantly higher than those in the monochromatic light treatments, the transpiration rate (*E*) and stomatal conductance (*G*_s_) were the highest in the B treatment, and the intercellular CO_2_ concentration (*C*_i_) was the highest in the Y treatment. The non-photochemical quenching coefficient (NPQ) was the highest in the Y treatment, but the other chlorophyll fluorescence characteristics differed among treatments in the following order: W > B > R > G > Y. This includes the maximum photochemical efficiency of photosystem II (PSII) under dark adaptation (Fv/Fm), maximum photochemical efficiency of PSII under light adaptation (Fv′/Fm′), photochemical quenching coefficient (qP), actual photochemical efficiency (ΦPSII), and apparent electron transport rate (ETR). Finally, the leaf structure and chloroplast ultrastructure showed the most complete development in the B treatment.

**Conclusions:**

White and blue light significantly improved the photosynthetic efficiency of Welsh onions, whereas yellow light reduced the photosynthetic efficiency.

## Background

Light is not only the primary source of energy for photosynthesis in plants but is also an important signal for plant growth [1]. Light intensity, quality, and photoperiod can regulate plant development and secondary metabolism [2–5]. Johkan [6] found that the net photosynthetic rate (*P*_n_) of *Lactuca sativa* leaves irradiated with green (G) light-emitting diodes (LEDs) at a photosynthetic photon flux (PPF) of 200 μmol/m^2^·s was significantly higher than that of plants grown under a PPF of 100 μmol/m^2^·s. The *P*_n_ of the plants irradiated with a G510 light (peak wavelength: 510 nm; bandwidth at half peak height: 18 nm) was the highest among all of the light sources [6]. Many plant processes are regulated by the wavelength of light experienced during growth, including seed germination, photomorphogenesis, photosynthesis, carbon and nitrogen metabolism, biomass accumulation, chloroplast ultrastructure, and leaf anatomical structure [7–12]. Studies have shown that the ratio of red (R) light to far-R light regulates the flowering time of *Arabidopsis*, providing evidence for the existence of wavelength-specific pathways in plant flowering times [13].

Photosynthesis is an important biological process for plant life, which has played an important role in the evolution of the Earth’s ecosystems. Increasing photosynthetic rates are critical for increasing crop yields to meet the rising demands for food [14, 15]. Chloroplast development and chlorophyll (Chl) metabolism are key components of photosynthesis in green plants, and previous studies have shown the existence of Chl synthesis-related enzymes that regulate chloroplast development [16, 17]. In cucumber seedlings, the photosynthetic rate was significantly higher under white (W) light than under red (R), blue (B), yellow (Y), or G light, and the morphology and photosynthetic rate differed significantly under the different monochromatic light treatments [18]. In tomato, lower height, biomass, and leaf area were noted for plants grown under RGB light (33% R, 33% G, and 33% B) and RB light (66% R and 33% B) than under W light [19]. Further, compared to W light growth conditions, monochromatic light also lowered the growth, *P*_n_, stomatal conductance (*G*_s_), intercellular CO_2_, and transpiration rates of tobacco plants [11]. Finally, in *Camptotheca acuminata* seedlings, R light promoted the development of chloroplasts and improved photosynthetic efficiency [20].

Ribose-1,5-bisphosphate carboxylase/oxygenase (RuBisCO; EC 4.1.1.39) is a key plant photosynthetic enzyme that controls carbon dioxide (CO_2_) and carbon fixation. The Calvin and photorespiration cycles are shunted by RuBisCO, and the relative magnitudes of their activities directly affect the photosynthetic rate [21]. Gao [22] found that when the ratio of R to B light was 4:1, the ribose-1,5-bisphosphate carboxylase (RuBPCase) activity in purple lettuce was significantly higher than that in other light treatments.

LEDs are widely used in horticultural facilities, and research into their effects on the growth and development of horticultural crops is of great interest. Previous studies have examined the relationships between light and growth, photosynthetic characteristics, carbon and nitrogen metabolism, and volatile production of other plants. Lin [23] showed that the root fresh weight (FW) and dry weight (DW) of lettuce were higher when treated with RBW light and full-spectrum light (FL) than RB light. Zhang [24] found that the sucrose, fructose, and glucose content of peach fruits grown under natural light was higher than that in fruits grown in environments covered with B, R, G, or Y film. Prior studies have also examined the responses of plant volatiles to different light conditions. For example, basil plants grown under BRY or BRG light showed high evaporation levels of monoterpenoid volatiles, while the same plants grown under far-infrared-B-R (far-RBR) light showed even higher evaporation levels of most sesquiterpenoid volatiles [25].

The Welsh onion (*Allium fistulosum* L.) is an important seasoning vegetable, for which the main flavor derives from an organic sulfide (a key indicator of nutritional quality) [26]. The organic sulfide content of the plant can be expressed as the pyruvic acid content, its decomposition product [27]. In recent years, the production of Welsh onions in industrial facilities has expanded, but few studies have investigated the light-induced effects on its growth, photosynthetic characteristics, and flavor. In this study, we examine the growth, photosynthetic characteristics, and leaf anatomy of Welsh onions grown under different wavelengths of light and provide a theoretical basis for the regulation of the light environments in industrial facilities.

## Results

### Growth and development of welsh onions under different light conditions

**Fig. 1:**
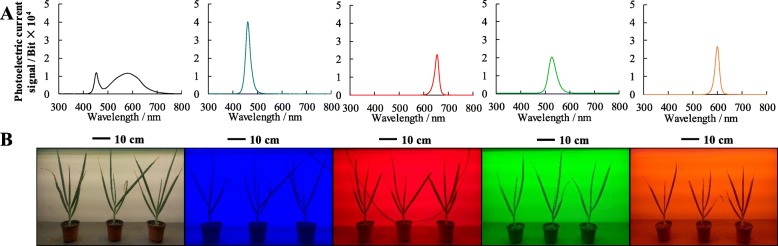
**a** Characteristics of the respective LED irradiance spectra in the different treatments; **b** the different LED light treatments tested

The leaf number, leaf area (LA), plant height, cauloid diameter, and cauloid FW of the Welsh onions were significantly higher after 30 days under W light than under any of the monochromatic light treatments. The growth of plants in the B light treatment was significantly higher than that under the other monochromatic light treatments. The shoot dry matter content of plants in the B treatment was slightly higher than that in plants in the W treatment, indicating a higher water content in the W light-grown plants. Dickson’s quality index (DQI) is an assessment of seedling quality and performance [28]; the DQI of seedlings in the monochromatic light treatments differed in the following order: B > R > G > Y (Fig. 1b, Table 1).

**Table 1:** Growth and development of Welsh onions under different light conditions

Light quality	Leaf number	Leaf area (cm2)	Plant height (cm)	Cauloid diameter (mm)	Leaf FW (g/plant)	Cauloid FW (g/plant)	Root FW (g/plant)	Shoot dry matter %	DQI
W	4.60 ± 0.55a	151.61 ± 2.40a	49.80 ± 1.26a	11.63 ± 0.43a	19.48 ± 0.78a	10.88 ± 1.55a	2.32 ± 0.10a	9.82 ± 0.17b	0.22 ± 0.01a
B	4.40 ± 0.55ab	142.60 ± 6.16b	47.44 ± 1.24b	9.66 ± 0.28b	14.34 ± 0.64b	9.04 ± 0.22b	1.83 ± 0.10b	10.08 ± 0.10a	0.16 ± 0.01b
G	3.40 ± 0.55c	114.15 ± 3.40d	47.44 ± 2.11b	8.04 ± 0.09d	12.07 ± 0.56c	7.49 ± 0.19c	1.66 ± 0.05c	9.01 ± 0.12d	0.13 ± 0.00d
Y	3.80 ± 0.45bc	99.60 ± 4.42e	44.44 ± 1.43c	8.29 ± 0.27d	11.47 ± 0.52c	7.24 ± 0.25c	1.55 ± 0.11c	8.97 ± 0.24d	0.11 ± 0.01e
R	4.20 ± 0.45ab	122.14 ± 1.73c	51.00 ± 1.91a	8.91 ± 0.17c	13.65 ± 0.77b	8.99 ± 0.43b	1.85 ± 0.07b	9.37 ± 0.10c	0.14 ± 0.01c

Values are means of 5 replicates ± standard deviation (SD). Different letters (a, b, c, d) in the same column indicate significant differences among treatments at *P* ≤ 0.05 according to Duncan’s new multiple range test. *W* white light, *B* blue light, *G* green light, *Y* yellow light, *R* red light. *n* = 5

### Photosynthetic pigment content of welsh onions under different light conditions

The Chl a and Chl b content in the W treatment were significantly higher than that in the monochromatic light treatments, and among the monochromatic treatments, the Chl content was the highest in the B treatment. The carotenoid content tended to be consistent with the Chl content (Table 2). Chlorophyll b plays an important role in plant adaptation to low-light conditions—in low light, plants synthesize more Chl b and increase their Chl a/b ratio, which helps to form a larger light-harvesting system [29]. The Chl a/b ratio was the lowest under G light and differed among the remaining treatments (although not significantly; see Table 2). These results suggest that, in addition to FL (W), B light may promote Chl a, Chl b, and carotenoid content in Welsh onions. These results also suggest that G light could enhance the absorption ability of Welsh onions in low-light conditions.

**Table 2:** Photosynthetic pigment content of Welsh onions under different light conditions

light quality	chlorophyll a (mg∙g^− 1^)	chlorophyll b (mg∙g^− 1^)	Carotenoid (mg∙g^− 1^)	chlorophyll a + b (mg∙g^− 1^)	chlorophyll a/b
W	0.61 ± 0.004a	0.27 ± 0.002a	0.20 ± 0.001a	0.88 ± 0.005a	2.23 ± 0.019b
B	0.54 ± 0.002b	0.23 ± 0.005b	0.17 ± 0.001b	0.77 ± 0.003b	2.34 ± 0.059a
G	0.44 ± 0.002d	0.20 ± 0.005d	0.15 ± 0.001d	0.65 ± 0.005d	2.16 ± 0.058c
Y	0.39 ± 0.004e	0.17 ± 0.004e	0.12 ± 0.001e	0.56 ± 0.003e	2.26 ± 0.067b
R	0.49 ± 0.001c	0.22 ± 0.002c	0.16 ± 0.001c	0.70 ± 0.003c	2.26 ± 0.024b

Values are means of 5 replicates ± SD. Different letters (a, b, c, d) in the same column indicate significant differences among treatments at *P* ≤ 0.05 according to Duncan’s new multiple range test. *W* white light, *B* blue light, *G* green light, *Y* yellow light, *R* red light. *n* = 5

### Photosynthetic parameters of welsh onions under different light conditions

**Fig. 2:**
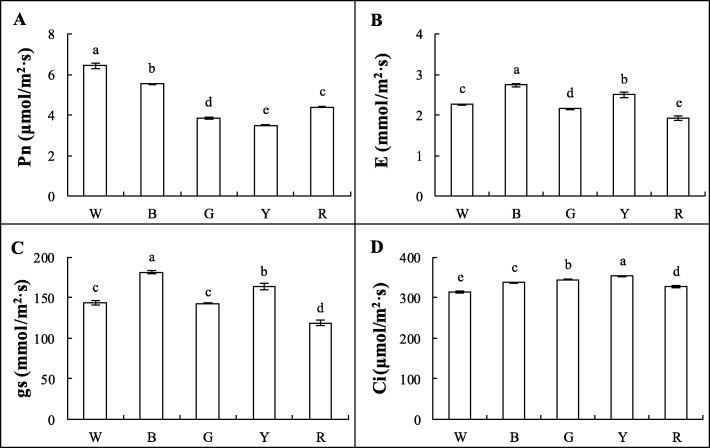
Photosynthetic parameters of Welsh onions under different light conditions, including: **a** net photosynthetic rate (*P*_n_); **b** transpiration rate (*E*); **c** stomatal conductance (*G*_s_); and **d** intercellular CO_2_ concentration (*C*_i_). These photosynthetic parameters were measured under white, blue, red, green, and yellow light for about 5 min each. Values are means of 5 replicates ± SD. Different letters (a, b, c, d) in the same column indicate significant differences among treatments at *P* ≤ 0.05 according to Duncan’s new multiple range test. W: white light; B: blue light; G: green light; Y: yellow light; R: red light. *n* = 5

The photosynthetic parameters were measured under W, B, R, G, and Y lights for approximately 5 min each. The *P*_n_ of Welsh onion leaves was the highest in the W treatment, and decreased in turn in the B, R, G, and Y treatments, respectively. On the 30th day of treatment, the leaf *P*_n_ was (W) 6.45 μmol/m^2^·s, (B) 5.54 μmol/m^2^·s, (G) 3.87 μmol/m^2^·s, (Y) 3.50 μmol/m^2^·s, and (R) 4.40 μmol/m^2^·s (Fig. 2a). The *G*_s_ and transpiration rate (*E*) were significantly higher in the B treatment than in the W and monochromatic light treatments, which decreased in turn for the Y, G, and R treatments (Fig. 2b-c). The intercellular CO_2_ concentrations (*C*_i_) in the Y and G treatments were significantly higher than those in the other treatments, and *C*_i_ was the lowest in the W treatment (Fig. 2d). These results show that B light could improve the photosynthetic gas exchange of Welsh onion plants.

### Chlorophyll fluorescence parameters of welsh onions under different light conditions

**Fig. 3:**
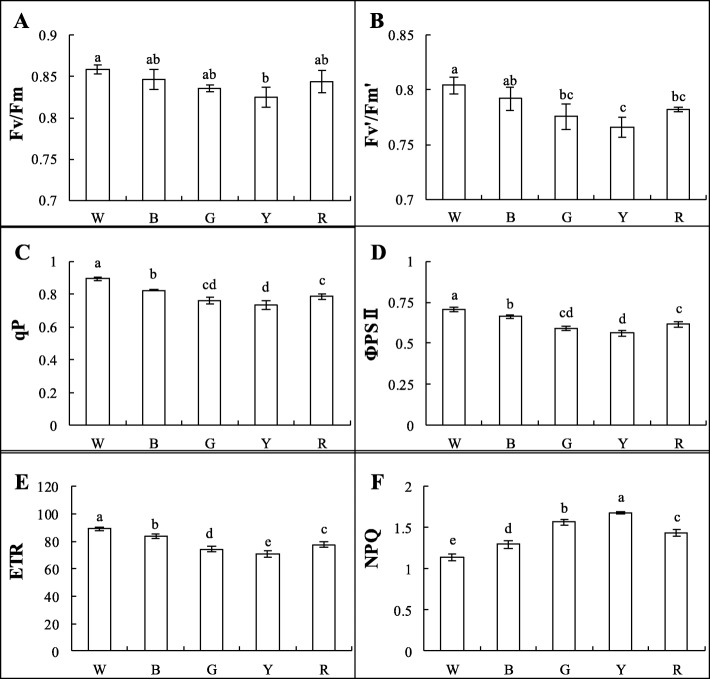
Chlorophyll fluorescence parameters of Welsh onions under different light conditions, including: **a** maximum photochemical efficiency of PSII under dark adaptation (Fv/Fm); **b** maximum photochemical efficiency of PSII under light adaptation (Fv’/Fm′); **c** photochemical quenching coefficient (qP); **d** actual photochemical efficiency (ΦPSII); **e** apparent electron transport rate (ETR); and **f** non-photochemical quenching coefficient (NPQ) = 1-(Fm′-Fo’)/(Fm-Fo). Values are means of 5 replicates ± SD. Different letters (a, b, c, d) in the same column indicate significant differences among treatments at *P* ≤ 0.05 according to Duncan’s new multiple range test. W: white light; B: blue light; G: green light; Y: yellow light; R: red light. *n* = 5

**Fig. 4:**
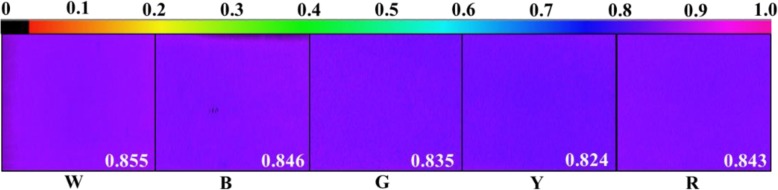
Chlorophyll fluorescence imaging analysis of Welsh onion leaves different light conditions, including the maximum photochemical efficiency of PSII under dark adaptation (Fv/Fm) in them

For all of the light treatments (except W), the maximum photochemical efficiencies of photosystem II (PSII) under dark adaptation (Fv/Fm) and light adaptation (Fv′/Fm′) were significantly higher than those in the Y treatment; no differences were noted for the other treatments (Figs. 3a-b and 4). The photochemical quenching coefficient (qP) was significantly higher in the W treatment than in the other treatments. Among the monochromatic light treatments, qP was the highest in the B treatment and the lowest in the Y treatment (Fig. 3c). The actual photochemical efficiency (ΦPS II) and apparent electron transport rate (ETR) showed consistent trends among the treatments with qP—both were significantly higher in the W treatment and decreased sequentially in the B, R, G, and Y treatments, respectively (Fig. 3d-e). The non-photochemical quenching coefficient (NPQ) and qP showed opposite trends: NPQ was the highest in the Y treatment and the lowest in the W treatment (Fig. 3f). These results indicate that B light could increase the proportion of the reaction centers in PSII opening under light adaptation, enhance PSII reaction center activity, and increase the electron transfer rate, while Y light could increase the heat dissipation of Welsh onion plants.

### Leaf anatomy and chloroplast ultrastructure of welsh onions under different light conditions

**Fig. 5:**
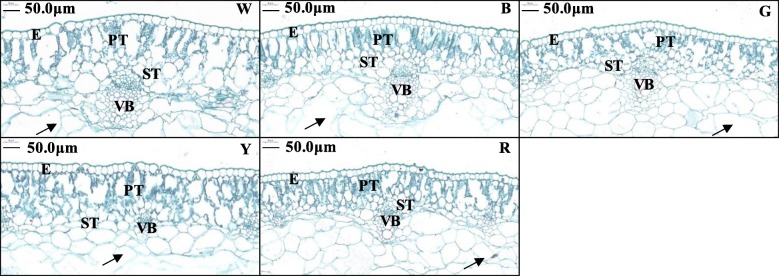
Leaf anatomy of Welsh onions under different light conditions. The fistular lamina of Welsh onion (*Allium fistulosum* L.) leaves changes from being solid to hollow during development, and the cells around the cavity break up until the remaining 1–2 layers of cells from the palisade layer show cell wall residues (‘arrowheads’) [30]. E: epidermis; PT: palisade tissue; ST: spongy tissue; VB: vascular bundle; W: white light; B: blue light; G: green light; Y: yellow light; R: red light. Scale bars = 50 μm

Welsh onion plants have fistular leaves; the fistular lamina change from solid to hollow during development, and the cells around the cavity break up until the remaining 1–2 layers of cells from the palisade show cell wall residues (i.e., “arrowheads”) [30]. Under B light, red–green staining of leaf slices revealed similarly sized and tightly arranged palisade tissue in each layer with dense Chl. This suggests that the spaces in the leaf could be used more efficiently to absorb light energy, thereby contributing to improved photosynthesis. On the contrary, the W-, R-, and G-treated leaves had relatively disordered palisade tissue cells, and in the Y treatment, the palisade tissue showed loose arrangement. Differences in leaf vascular bundle sizes under the different light treatments were observed in the following order: W > B > R > G > Y. There were no significant differences in the spongy mesophyll tissue thickness or arrangement among the treatments (Fig. 5).

**Fig. 6:**
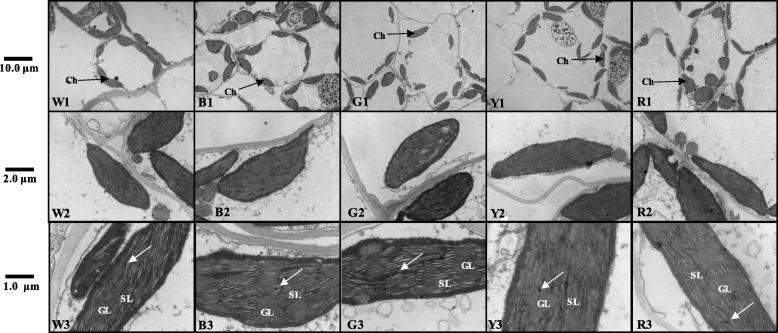
Chloroplast ultrastructure of Welsh onions under different light conditions. Transmission electron microscopy observations of mesophyll cells in Welsh onion leaves exposed to W Light (**w1**-**w3**) and B, G, Y, R Light (**b1**-**r3**). The bars shown are 10 μm, 2 μm, 1 μm, respectively. The size and arrangement density of chloroplasts could be clearly seen at 10 μm and 2 μm, and the grana lamella and stroma lamella of chloroplasts could be clearly seen at 1 μm. Ch: chloroplast; GL: grana lamella; SL: stroma lamella; white arrow: osmiophilic particles; W: white light; B: blue light; G: green light; Y: yellow light; R: red light

Chloroplasts contain Chl and are the sites of photosynthesis in plant cells. If Chl synthesis is reduced or blocked, the chloroplast structure will change [31]. The different light treatments used in this study greatly affected the chloroplast development in the leaves of Welsh onion plants. The size and shape of the chloroplasts in the mesophyll cells were observed by transmission electron microscopy after sampling on the 30th day of treatment. Normally, the chloroplasts are fusiform or elliptically shaped and are arranged along the plasma membrane. The structure of the granular sheets (grana lamellae) is also visible and runs parallel to the long axis, and the thylakoids are closely packed and arranged in a neat and orderly manner. Chloroplasts such as this were observed in the W and B treatments (Fig. 6w1-b3). The thylakoid membranes in the B-treated leaf cells grew the most, suggesting greater light-capturing ability and improved energy conversion efficiency of the photosynthetic membrane [32]. The chloroplasts of the Y-treated leaves were smaller, and the granular lamellae of the thylakoids were degraded, suggesting decreased photosynthetic capacity (Fig. 6y1-y3). Our results indicate that the chloroplasts of leaves treated with B and R lights were intact and contributed to photosynthesis, while Y light inhibited chloroplast development.

### RuBPCase activity of welsh onions under different light conditions

**Fig. 7:**
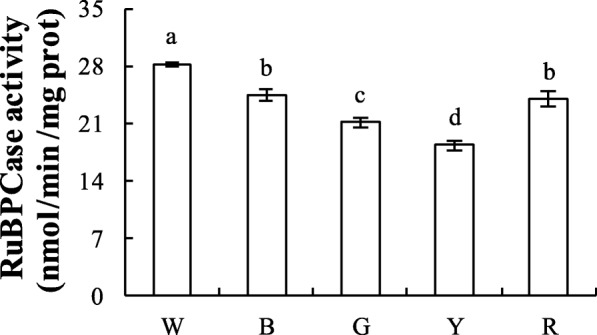
RuBPCase activity of Welsh onions under different light conditions. The ribulose-1,5-bisphosphate carboxylase (RuBPCase) activity of RuBisCo was determined using an ELISA kit (Suzhou Keming). Values are means of 5 replicates ± SD. Different letters (a, b, c, d) in the same column indicate significant differences among treatments at *P* ≤ 0.05 according to Duncan’s new multiple range test. W: white light; B: blue light; G: green light; Y: yellow light; R: red light. *n* = 5

RuBPCase is a key enzyme for photosynthesis, and the different light treatments had a significant effect on RuBPCase activity. The activities of RuBPCase were 28.21, 24.39, 20.91, 18.31, and 23.57 nmol/(min·mg prot) in the W, B, G, Y, and R treatments, respectively. Among the different monochromatic light treatments, the B treatment increased RuBPCase activity, while the Y treatment inhibited RuBPCase activity (Fig. 7). These results suggest that B light could improve RuBPCase activity, which would affect the *P*_n_ of Welsh onion plants.

## Discussion

The light environment that crops are grown in is one of the most important environmental factors affecting plant growth and development. Light is the driving force behind photosynthesis [33], the most important chemical reaction on Earth, and the light intensity and spectral composition act as signaling factors during plant development [34]. The visible light spectrum ranges from 380 to 780 nm, whereas shorter wavelengths (< 380 nm) represent ultraviolet light, and longer wavelengths (> 780 nm) represent infrared light. In nature, the spectral composition of the light experienced by plants is constantly changing; there is more B light during the day, more R light in the morning and evening, and a strong influx of W light at noon (weather introduces even more variation) [35]. Under a plant canopy, the spectral composition of the light also changes, which can be used as an important signal for the light competition response in plants. Physiological studies have shown that plants use their photoreceptors to perceive the composition of light reflected from the surrounding environment [36] and use this information to accurately predict of future competition, even to the point of inducing morphological responses to avoid light competition before direct shading occurs [37].

In this study, there were significant differences in the growth and development of Welsh onion plants grown under different wavelengths of LED light (Table 1). We found that the full-spectrum, W-treated plants grew best, which is consistent with the conclusions of previous studies on cucumbers and *Cyclocarya paliurus* [18, 38]. Among the monochromatic light treatments, the plants grown in B light were more compact, whereas those grown in G and Y light were not as compact (Fig. 1b). This may have been due to the responses of the leaves to different optical signals, which is consistent with the results of previous studies on rice and lettuce [39, 40]. However, studies have shown different results for *Camptotheca acuminata* Decne. seedlings, wheat, and other crops [9, 41], suggesting possible species-specific variation. In cucumber seedlings, the growth rate and LA were reduced as the proportion of B light in the environment decreased; the plant growth rate was the lowest in 100% R light (0% B) and the highest in 100% B light (0% R), which may be owing to a variety of morphological and physiological factors [42].

Previous studies have shown that photosynthetic pigments can absorb and transmit light energy. Light affects the synthesis of photosynthetic pigments, which affects photosynthesis, and thus, plays an important role in plant growth and development [43, 44]. Chlorophyll absorbs light energy and transfers it to the chloroplasts for photosynthesis [45]. In our study, the Chl content and its ratio were significantly affected by the different light treatments (Table 2). The total Chl content decreased under monochromatic light, indicating that monochromatic light causes damage to the photosynthetic pigments. However, compared to R light, B light treatment resulted in significantly higher leaf Chl content, which is inconsistent with the results of studies on lettuce and *Anoectochilus roxburghii* [46, 47]. In this study, the *P*_n_ of Welsh onion seedlings was significantly lower when grown under monochromatic light than under the W light treatment, especially under Y and G lights (Fig. 2a). This is consistent with the results from cucumber and *Acacia mangium* seedlings [48, 49]. Our results also show that the *P*_n_ was significantly associated with the Chl and RuBisCO content. The *E* and *G*_s_ were the highest under B light (Fig. 2b-c), probably because the stomata of the seedlings exposed to B light were well-developed, suggesting that the nitrogen accumulation of the B-treated plants was higher than that under the other monochromatic light treatments [50].

RuBisCO, acting as RuBPCase, is a key enzyme in the Calvin cycle, the nature of which determines photosynthetic efficiency and productivity. Previous studies have shown that light affects the RuBPCase activity of algae [51, 52]. In our study, the high photosynthetic rate in B light may have been related to the high Chl content (Table 2), light energy conversion efficiency (Fig. 3b), and RuBPCase activity of the plants in this treatment (Fig. 7).

The Chl fluorescence of green plants reflects their photosynthetic potential in a complex manner [53]; green plants absorb light, some of which is used for photosynthesis, some is re-emitted as Chl fluorescence, and some is used for heat dissipation [54]. Fv/Fm, ETR, and qP are parameters for the maximum photochemical efficiency of PSII and the ratio of reaction centers in PSII that are oxidized (open), which are indicators of photosynthetic efficiency. NPQ is a parameter that indicates the ability of chloroplasts to dissipate excess excitation (light) energy as superheat [55]. ΦPSII is often used to indicate the quantum yield of electron transfer during plant photosynthesis, reflecting the actual light energy capture rate when the reaction centers are partially closed. Among the monochromatic light treatments used in this study, the Fv/Fm, ETR, qP, and ΦPSII values were the highest in the B-treated plants, but NPQ was lower (Fig. 3). Therefore, we propose that B light increased the rate of photosynthesis in Welsh onion plants but reduced the heat dissipation ability of PSII. Thus, B light is beneficial to Welsh onions because it improves the efficiency of light energy conversion and allows increased energy accumulation for carbon assimilation in the dark reactions (Fig. 3a-b). In a similar study, *Phalaenopsis* had lower Fv/Fm values in 100% R (0% B) light than in treatments containing more B light [56].

The anatomical structure of the leaves was significantly changed by the different LED light treatments. In the W treatment, we observed loosely arranged palisade mesophyll cells with large spaces (Fig. 5w), which is consistent with the results of a study of potato (*Solanum tuberosum* L.) leaves [57]. Monochromatic light did not influence leaf thickness, but in cucumber leaves, R light severely reduced the thickness of the leaf fence and sponge tissue [58]. Sæbø [59] in vitro cultured *Betula pendula* Roth under different light conditions and found larger epidermal cell areas under B light and smaller areas under R light. These comparisons support the hypothesis that there are species-specific responses to the light environment.

Chloroplasts are the major photosynthesis organelles and are rich in thylakoid membranes that absorb light energy and transport and transform it during photosynthesis [60]. The composition of light greatly influences the ultrastructure of chloroplasts and the thylakoid membrane [61, 62]. In this study, the number of chloroplasts per cell and the number of grana lamellae in each chloroplast (on day 30) were the highest in plants grown under B light (Fig. 6w1-b3). This is consistent with the results of a study on cucumber leaves [63]. Upland cotton (*Gossypium hirsutum* L.) seedlings grown under B LEDs also showed high integrity of the chloroplast ultrastructure with a clearly visible lamellar structure Li [64]. This may be related to the expression of several chloroplast-encoded genes that require B light [65]. Our results show that the chloroplast membrane structure under B light is similar to the W light treatment, which is consistent with the results of a study on barley leaves [66]. However, in the study, the number of thylakoid membranes and the length of the extended accumulation zone in the chloroplasts in the R light treatment increased significantly. The grana also had a large diameter, irregular shape, and many prominent thylakoids. In some areas, the thylakoids were disordered [66]. This was not the case for the chloroplasts of the R-treated Welsh onions in this study; the chloroplasts were relatively intact, which may reflect differences based on species.

## Conclusions

The growth and development of plants is strongly influenced by the spectrum of light in their environment. Here, we investigated the growth, photosynthetic characteristics, and chlorophyll fluorescence characteristics of Welsh onions grown under different wavelengths of LED light, to reveal the effects of light on photosynthesis and to provide a theoretical basis for the regulation of the light environments used in production facilities.

As expected, the growth and morphology of Welsh onion plants were altered by the different light spectra, and the light treatments significantly affected the photosynthesis-related processes. The full-spectrum, white light treatment was the most beneficial for plant growth. Among the monochromatic light treatments, the chlorophyll content, chlorophyll a/b ratio, net photosynthetic rate, stomatal conductance, and transpiration rate were the highest in the blue light treatment, indicating that blue light is beneficial to photosynthesis in Welsh onion. Changes in the leaf structure suggest that red light may play an important role in chloroplast development and delaying leaf senescence. However, the yellow light treatment induced NPQ, which affected plant morphology, destroyed leaf tissue and thylakoid membrane structure, reduced the photosynthetic pigment content, and significantly reduced the net rate of photosynthesis. In summary, different monochromatic light spectra were found to play unique roles in the growth and photosynthesis of the Welsh onion.

## Methods

### Materials and treatments

The experiment was carried out in the light quality culture room at the College of Horticulture Science and Engineering, Shandong Agricultural University, Shandong, China (longitude: 117.12°E; latitude: 36.19°N) during October and November 2018. We used the Welsh onion variety ‘Yuanzang’, which was originally sourced from the Tai’an Taishan Seed Industry Technology Co., Ltd. The seeds were plants in 50-hole trays, and the cultivation substrate was a 6:3:1 mixture of charcoal, perlite, and vermiculite. The seedlings were watered with 1/2 Hoagland nutrient solution every 3 days after sowing. When the seedling height was approximately 5 cm, the plants were thinned so that only one seedling per hole remained. When the seedling height was approximately 15 cm, 2–3 pieces of leaves were sampled and placed in the LED light treatments. The light treatments used dimming plant lamps (Huizhou Kedao Technology Co., Ltd.) of five different wavelengths: W light (control group), B light, G light, Y light, and R light. The spectral characteristics of the LED sources were measured with a UNSPEC-DCTM spectrum analyzer (PP-SYSTEMS, UK), with a bandwidth of 300–1100 nm and a 3.3 nm scanning interval. The spectral characteristics of each light treatment are shown in Fig. 1a-b.

The light intensity was maintained at 301.6 ± 12.7 μmol/m^2^·s. The day/night temperature was maintained at 25 °C/18 °C, respectively, the relative humidity was 65.2 ± 4.5%, and the light/dark (L/D) photoperiod was set to 12 h/12 h. Each treatment contained 20 plants, and all treatments and assays were repeated 5 times.

### Measurement of morphological and physiological characteristics

The Welsh onion plants grown in different light treatments were randomly sampled and measured 30 days after planting. The measurements included the leaf number, LA, plant height, cauloid diameter, leaf FW, cauloid FW, root FW, and aboveground dry matter content. The plant height and cauloid diameter were measured with a ruler and Vernier caliper, respectively. The LA was determined using a LI-3000C leaf area meter (LI-COR Biosciences, USA). For the biomass measurements, the samples were divided into two parts: the shoot and the roots. The two parts were placed in a box, dried at 75 °C for 48 h, and then weighed for the shoot and root DW, total DW, and root/shoot ratio in dry weight basis (R/S). From these measures, we calculated DQI as follows [28]:

$$ \mathrm{Dickson}'\mathrm{s}\;\mathrm{Quality}\ \mathrm{Index},\mathrm{DQI}=\frac{\mathrm{Seedling}\;\mathrm{dry}\;\mathrm{weight}\;\left(\mathrm{g}\right)}{\frac{\mathrm{Height}\;\left(\mathrm{cm}\right)}{\mathrm{Root}\kern0.17em \mathrm{collar}\kern0.17em \mathrm{diameter}\;\left(\mathrm{mm}\right)}+\frac{\mathrm{Shoot}\;\mathrm{dry}\;\mathrm{weight}\;\left(\mathrm{g}\right)}{\mathrm{Root}\;\mathrm{dry}\;\mathrm{weight}\;\left(\mathrm{g}\right)}}. $$

### Measurement of photosynthetic pigment content

The Chl content of the leaves was determined by 80% acetone extraction. A fresh 0.2 g sample of the third leaf blade was weighed and placed in a 20 mL test tube containing 5 mL of absolute ethanol and 5 mL of 80% acetone and incubated in darkness for 24 h. The optical density (OD) was measured using a UV-1200 spectrophotometer (Shimadzu, Japan) at 470 nm (OD_470_) for carotenoids, 663 nm (OD_663_) for Chl a, and 645 nm (OD_645_) for Chl b. These measurements were used to calculate the content of each respective pigment in the leaves using the following formulas [67, 68]:

$$ {\displaystyle \begin{array}{l}\mathrm{Chla}\left(\mathrm{mg}\kern0.28em \cdot {\mathrm{g}}^{-1}\right)=\left(12.72{\mathrm{OD}}_{663\mathrm{nm}}-2.59{\mathrm{OD}}_{645\mathrm{nm}}\right)\mathrm{V}/1\kern0.28em 000\kern0.28em \mathrm{W};\\ {}\mathrm{Chlb}\left(\mathrm{mg}\kern0.28em \cdot {\mathrm{g}}^{-1}\right)=\left(22.88{\mathrm{OD}}_{645\mathrm{nm}}-4.67{\mathrm{OD}}_{663\mathrm{nm}}\right)\mathrm{V}/1\kern0.28em 000\kern0.28em \mathrm{W};\\ {}\mathrm{Carotenoids}\left(\mathrm{mg}\kern0.28em \cdot {\mathrm{g}}^{-1}\right)=\left(1\kern0.28em 000{\mathrm{OD}}_{470\mathrm{nm}}-3.27\mathrm{Chla}-104\mathrm{Chlb}\right)\mathrm{V}/\left(229\times 1000\kern0.28em \mathrm{W}\right),\end{array}} $$

where V is the total volume of acetone extract (ml), and W is the FW (g) of the sample.

### Measurement of photosynthetic characteristics and chlorophyll fluorescence

On day 30 (after planting), the functional third leaves of the plants were sampled and *P*_n_, *G*_s_, *C*_i_, and *E* were measured using a Li-6800 portable photosynthetic apparatus (Li-COR, USA) following the methods of Li [69], with slight modifications. To measure the CO_2_ fixation by photosynthesis under different light conditions, the gas exchange characteristics of the functional leaves were measured under each light source. The leaf chamber temperature and leaf CO_2_ concentration were maintained at 25 °C and 400 μmol/m^2^·s, respectively, and the vapor pressure deficit in the leaf chamber was kept at 1.0 kPa. When the *P*_n_ reached steady state (after about 5 min), it was recorded. The measurements were repeated 5 times for each light treatment, and the average value was calculated for each photosynthetic parameter. The RuBPCase activity of RuBisCO was determined using an enzyme-linked immunosorbent assay kit (Suzhou Keming).

The Chl fluorescence of the third fully expanded functional leaf was measured using an M-series modulated Chl fluorescence imaging system (MINI-IMAGING-PAM, Walz, Effeltrich, Germany). To do so, the fluorescence parameters were first determined after dark adaptation for 20 min. Initial fluorescence (Fo) was measured after induction by a weak modulation (0.05 μmol/m^2^·s), followed by excitation with a strong saturation pulse (6000 μmol/m^2^·s, pulse time = 2 s) to produce and measure the maximum fluorescence (Fm). Next, for light adaptation, the Fo and Fm′ (the maximum fluorescence yield obtained when the light-adapted sample was exposed to the saturation pulse) were directly measured under each LED light before the actinic light was turned on, followed by a series of saturation pulses under each LED light. Multiple strong saturated flash pulses were applied (6000 μmol/m^2^·s, pulse time = 2 s), and the fluorescence yield (Ft) and Fm′ under light adaptation with each LED light were measured every 20 s until pulse termination. We calculated the average values of the last six flashes (after a substantially steady state was reached after 10 flashes), and the measurements from five plants were averaged for each treatment. The measured indicators included Fo, Fm, and Ft. Other fluorescence parameters were calculated according to Genty [70]:

$$ {\displaystyle \begin{array}{l}\mathrm{Maximum}\ \mathrm{photochemical}\ \mathrm{efficiency}\ \mathrm{of}\ \mathrm{photosystem}\ \mathrm{II}\left(\mathrm{PSII}\right)\mathrm{under}\ \mathrm{dark}\ \mathrm{adaptation}\left(\mathrm{Fv}/\mathrm{Fm}\right)=\left(\mathrm{Fm}-\mathrm{Fo}\right)/\mathrm{Fm};\\ {}\mathrm{Maximum}\ \mathrm{photochemical}\ \mathrm{efficiency}\ \mathrm{of}\ \mathrm{PSII}\ \mathrm{under}\ \mathrm{light}\ \mathrm{adaptation}\left(\mathrm{Fv}^{\prime }/\mathrm{Fm}^{\prime}\right)=\left(\mathrm{Fm}^{\prime }-\mathrm{Fo}^{\prime}\right)/\mathrm{Fm}^{\prime };\\ {}\mathrm{Actual}\ \mathrm{photochemical}\ \mathrm{efficiency}\left(\varPhi \mathrm{PSII}\right)=\left(\mathrm{Fm}^{\prime }-\mathrm{Fs}\right)/\mathrm{Fm}^{\prime };\\ {}\mathrm{Non}-\mathrm{photochemical}\ \mathrm{quenching}\ \mathrm{coefficient}\left(\mathrm{NPQ}\right)=1-\left(\mathrm{Fm}^{\prime }-\mathrm{Fo}^{\prime}\right)/\left(\mathrm{Fm}-\mathrm{Fo}\right);\\ {}\mathrm{Photochemical}\ \mathrm{quenching}\ \mathrm{coefficient}\left(\mathrm{qP}\right)=\left(\mathrm{Fm}^{\prime }-\mathrm{Ft}\right)/\left(\mathrm{Fm}^{\prime }-\mathrm{Fo}^{\prime}\right);\\ {}\mathrm{Apparent}\ \mathrm{ETR}=\varPhi \mathrm{PSII}\cdotp \mathrm{PAR}\cdotp 0.5\cdotp 0.84,\mathrm{where}\ \mathrm{PAR}\ \mathrm{is}\ 300\;\mu \mathrm{mol}/{\mathrm{m}}^2\cdotp \mathrm{s}.\end{array}} $$

### Observation of the leaf anatomy and chloroplast ultrastructure of welsh onion

On day 30, paraffin sections (5 mm × 5 mm) of the samples were taken, fixed with a formalin–acetic acid–alcohol fixative, dehydrated in an alcohol and xylene series, embedded in paraffin, cross-sectioned to a thickness of 10 μm, and red–solid green stained. The total thickness of the transverse sections, as well as the thickness of the upper epidermis, palisade mesophyll tissue, and spongy mesophyll tissue, was measured under a light microscope using a micrometer.

Pieces of the functional leaves were sampled (1 mm × 1 mm), quickly placed in a 2.5% glutaraldehyde fixative solution, and evacuated with a vacuum pump. After the pieces sank to the bottom of the fixative solution, they were maintained at room temperature (25 °C) for 2 h, and then transferred to a refrigerator and stored at 4 °C. The samples were rinsed three times with 0.1 M phosphate buffer (PB, pH = 7.4) for 15 min each, fixed with 1% citric acid in 0.1 M phosphate-buffered saline (pH = 7.4) at room temperature (25 °C) for 5 h, and rinsed again three times with 0.1 M PB (pH = 7.4) for 15 min each. The leaf tissue was sectioned on a dehydration-infiltration-embedding-slicer (Leica, LeicaUC7) and imaged using a section-staining-transmission electron microscope (HITACHI, HT7700).

### Data analysis

All plants were randomly sampled in this study. The data were processed, plotted, and statistically analyzed in Excel 2016 and DPS software. The differences among treatments were tested using Duncan’s new multiple range test at a significance level of *P* ≤ 0.05.

## Supplementary information

**Additional file 1 Table S1.** Growth and development of Welsh onions under different light conditions. **Table S2.** Photosynthetic parameters of Welsh onions under different light conditions and RuBPCase activity of Welsh onions under different light conditions. **Table S3.** Chlorophyll fluorescence parameters of Welsh onions under different light conditions. **Table S4.** Leaf anatomy and chloroplast ultrastructure of Welsh onions under different light conditions.

## Data Availability

All data generated or analysed during this study are included in this published article and its additional files.

## Abbreviations

Ch: Chloroplast
Chl a: Chlorophyll a
Chl b: Chlorophyll b
*C*_i_: Intercellular CO_2_ concentration
DQI: Dickson’s Quality Index
E: Epidermis
E: Transpiration rate
ETR: Apparent electron transport rate
Fm: Maximum fluorescence
Fo: Initial fluorescence
Fv/Fm: Coefficient maximum photochemical efficiency of photosystem II under dark adaptation
Fv′/Fm′: Maximum photochemical efficiency of PSII under light adaptation
GL: Grana lamella
*G*_s_: Stomatal conductance
NPQ: Non-photochemical quenching
*P*_n_: Net photosynthetic rate
PSII: Photosystem II
PT: Palisade tissue
qP: Photochemical quenching coefficient
SL: Stroma lamella
ST: Spongy tissue
VB: Vascular bundle
ΦPSII: Actual photochemical efficiency
